# Supplementary material for: Patterns of Intron Gain and Loss in Fungi
Source: PLoS Biol. 2004 Nov 30;2(12):e422. doi: 10.1371/journal.pbio.0020422 (PMC532390; doi:10.1371/journal.pbio.0020422)
Supplement: Table S1 — Also available at http://genes.mit.edu/NielsenEtAl/. (4.3 MB ZIP). [file pbio.0020422.st001.zip › NielsenEtAl/html/1274.html]

AN7047.1.NCU07988.1.MG07075.1.FG01933.1


```
 CLUSTAL W (1.82) Multiple Sequence Alignments - Introns Inserted


Sequence 1: MG07075.1	424 aa
Sequence 2: FG01933.1	395 aa
Sequence 3: NCU07988.1	414 aa
Sequence 4: AN7047.1	410 aa
Alignment Length: 427 aa
Number Identitical Residues: 198 aa
Alignment Score (without introns) 9064


MG07075.1 	-MADRKRATDYFIDIVVFAGM0TATLYLLTKNILSSLSSTLGDPDKEKHEQARLRAKANL
NCU07988.1	-MTDNKRVAKDIMSGLLLAAT0TISVYFIFRDYVAPHVAKLIDPDRAKHDQNRLKAQQNL
FG01933.1 	-MGERRKLGDVAMDILLFAGM0MTAGLYVARNFLNPILSNIADPDKEKHEQARRQAKAHL
AN7047.1  	MASARSPRWQQIQDLIMIAGT0SATAYFLVRHLLSRLD---FDPDRQKKEEQQRKSAAIL
          	    .    .   . :::*.    :   : :. :        ***: *::: : ::   *

MG07075.1 	QRLRKIQNGDGTANGEAQDGEG--GQRNGPAVEELQLNEYENMIAMDVVAPEDINVGFDA
NCU07988.1	ARIQKSRDKSKVANDEFADDEVPEDSPSTVRVEDLILNEYESQVALEVVAPEDIPVGFDD
FG01933.1 	ERMNRNKR-DGLDYGDDSSD-----SRRGPRPEELVLNEYENLVALEMVPPEDISVGFDD
AN7047.1  	RKLDGNLDENSSGE-----------SGGKTRKGDLVLNQYEQAIAMDVVAPEDIPVSFKD
          	 ::      .               .       :* **:**. :*:::*.**** *.*. 

MG07075.1 	1IGGLEDIIEEVKESVIYPLTMPHLYSHAAPLLSAPSGVLLYGPPGCGKTMLAKAVAHES
NCU07988.1	~IGGLDEIIEEVREAIIYPLTMPQLYSHGGTLLSAPSGVLLYGPPGCGKTMLAKAVAHES
FG01933.1 	1IGGLDTIIEELKESIIYPLTMPHLYSHAAPLLSAPSGVLLYGPPGCGKTMLAKAVAHES
AN7047.1  	~IGGLEDIIEELKESVIYPLTMPHLFSSTSSLLTAPSGVLLYGPPGCGKTMLAKALAHES
          	 ****: ****::*::*******:*:*  ..**:*********************:****

MG07075.1 	GASFINLHISTLTEKWYGDSNKLVRAVFSLARKLQPAIIFIDEIDAVLGTRRSGEHEASG
NCU07988.1	GASFINLHISTLTEKWYGDSNKLVRAVFSLALKLQPAIIFIDEIDAVLGTRRSGEHEASG
FG01933.1 	GASFINLHISTLTEKWYGDSNKIVRAVFSLARKMQPAIIFIDEIDAVLGTRRSGEHEASG
AN7047.1  	GASFINLHISTLTEKWYGDSNKLVNAVFSLARKLQPSIVFIDEIDAVLGTRRSGEHEASG
          	**********************:*.****** *:**:*:*********************

MG07075.1 	MVKAE2FMTLWDGLTSANASGVPSRIMVLGATNRINDIDEAILRRMPKKFPVPLPGTEQR
NCU07988.1	MVKAE2FMTLWDGLTSTNSLGQPARIMVLGATNRINDIDDAILRRMPKKFPVPLPGKDQR
FG01933.1 	MVKAE2FMTLWDGLTSSNSSGMPARIMVLGATNRINDIDEAILRRMPKKFPVTLPGTEQR
AN7047.1  	MVKAE~FMTHWDGLTSANSLGEPQRVVVLGATNRMQDIDEAILRRMPKKFPVELPPAAQR
          	***** *** ******:*: * * *::*******::***:************ **   **

MG07075.1 	RRILELILGETKRDPEHFDLEYIAAVTAGMSGSEIKDACRDAAMSPMREFIRTQRAAGTP
NCU07988.1	RRILELVLADTKRDPE-FDLEYIAMVTEGMSGSELKEACRDAAMVPMREAIRNQKAAGKS
FG01933.1 	RRILQLVLQDTKTDPEHFNLDYVSRITAGLSGSDIKEACRDAAMVPVREYMRQHRESGKA
AN7047.1  	LGILSLVLKDTKIDRQNFDLHYLVKAMAGMSGSDIKEACRDAAMVPVREFIRQKTAQGLK
          	  **.*:* :** * : *:*.*:     *:***::*:******* *:** :* :   *  

MG07075.1 	MASIDPTQVRGIRTDDFFSRKGAGPRVRSMGENKKSTAKQNSTTEKHAERAEDSEYEDVD
NCU07988.1	IKKLDPNTVRGLRTNDFFGRKG--------GAPSLAADSRPSSVRPEAAMSEREIYEVET
FG01933.1 	MSTVDPKQFRGIRSDDFLGREG--------GQIKMQSTRQTSGVEELIAEDQD-------
AN7047.1  	ITSVDPSEVRGLRTEDFFASAGG--AQRFIPVATQNIPPETSSEKEWSTEDEAASENGTQ
          	: .:**. .**:*::**:.  *.          .     . *  .      :    .   

MG07075.1 	EEHTRPVSPD
NCU07988.1	ETEDVLS---
FG01933.1 	----------
AN7047.1  	QSRTTQAEA-
          	.      ..
```
